# Corticosteroids for Graves' Ophthalmopathy: Systematic Review and Meta-Analysis

**DOI:** 10.1155/2018/4845894

**Published:** 2018-11-22

**Authors:** Xiaofang Tu, Yan Dong, Hongmei Zhang, Qing Su

**Affiliations:** Department of Endocrinology, Xinhua Hospital, School of Medicine, Shanghai Jiaotong University, Shanghai, China

## Abstract

**Background:**

Graves' ophthalmopathy (GO) is a complicated autoimmune disease. Various therapies have been used to manage GO; however the optimum therapy is not clear. Glucocorticoids (GCs) therapy is the mainstay of treatment especially for active moderate to severe patients, which needs evidence-based support.

**Method:**

We searched all the randomized controlled trials (RCTs) involving corticosteroid treatment for patients diagnosed with GO from EMBASE, Medline, and the Cochrane library and then conducted a system review and meta-analysis. The electronic search covered the period from April 1966 to March 2018.

**Result:**

Twenty-nine trials were included. GCs were proved to be beneficial for GO patients [response rate, risk ratio (RR) = 1.72, 95% confidence interval (CI): 1.28~2.31, P=0.0003], and intravenous corticosteroids worked significantly better than oral corticosteroids as ever reported. When compared with the single treatment of GCs, the combination of radiotherapy and GCs showed similar effects on response rate (RR=1.25, 95%CI: 0.91~1.73). A study proved the advantage of mycophenolate mofetil over GCs in three outcomes (response rate, RR=0.74, 95%CI: 0.63~0.88). Additional treatments such as technetium-99 methylene diphosphate (^99^Tc-MDP) or cyclosporine enhanced the effect of GCs on proptosis reduction, respectively (P<0.00001 and P=0.02).

**Conclusion:**

Our meta-analysis confirmed the effects of GCs in the management of GO and intravenous GCs are proved to be better than oral GCs as ever reported. Combination of radiotherapy and GCs did not enhance the effects of GCs. However, if proptosis is the main issue, combination of ^99^Tc-MDP or cyclosporine with GCs may be taken into consideration. The reported advantages of mycophenolate mofetil over GCs are noteworthy and need more RCTs to confirm.

## 1. Introduction

Graves' ophthalmopathy (GO), or thyroid eye disease (TED), is regarded as an autoimmune disorder closely related to Graves's disease (GD). It may cause ocular symptoms including periorbital edema, chemosis, eyelid retraction, proptosis, altered ocular motility, and even diplopia, exposure keratopathy, and dysthyroid optic neuropathy (DON), which may result in visual loss. The prevalence rate of GO ranges from 0.1% to 0.3% [1].

The pathogenesis of GO is still not exactly known. It is difficult to assess and manage this complicated disease. Drug therapy, radiotherapy, and eye surgery have been used to improve the symptoms according to the activity and severity of GO. Lymphocytes and inflammation may play an important part in the pathogenesis. Thus, immunosuppression therapy, especially glucocorticoids (GCs), had become the mainstay of treatment for patients with active GO, which was also recommended by the European Group on Graves' Orbitopathy (EUGOGO) [2].

However, more detailed evidences were needed to support GCs as first-line treatment of GO. Therefore, we conducted a meta-analysis to compare the efficacy of GCs with other treatments for patients diagnosed with GO and to explore the ideal treatment regimen of GCs.

## 2. Materials and Methods

### 2.1. Data Source and Search Strategy

We searched randomized controlled trials (RCTs) from EMBASE, Medline, and the Cochrane library online according to a broad search strategy (S1 Strategy). The strategy included all the RCTs relevant to the glucocorticoid treatment including the monotherapy or the combined therapy with irradiation or other drugs for Graves' ophthalmopathy referring to some protocols from previous meta-analysis [3–5] and Cochrane Handbook for Systematic Reviews of Interventions. A manual search was done if necessary. The electronic search covered the period from April 1966 to March 2018.

### 2.2. Outcome Measures

The primary outcome was the response rate (i.e., the ratio of responders to a total number of patients) defined in each study. In addition, clinical activity score (CAS) and proptosis were also recorded to assess the therapeutic effects on the eye functions.

### 2.3. Trial Selection

Two reviewers assessed the eligibility of the studies independently based on the following predetermined selection criteria: (1) study design: randomized, controlled clinical trials; (2) population: patients diagnosed with GO; (3) intervention: at least one treatment for the GO was relevant to the glucocorticoid. The studies, which compared the operative treatment with drug therapy, were not included; (4) outcome variables: at least reporting one of the three outcomes mentioned above (i.e., response rate, CAS, and proptosis). The duplicate studies were moved. Any disagreement was solved by discussing or asking the third author.

### 2.4. Data Extraction

Two independent authors extracted the data from trials, respectively, by a customized form and then checked together. The following data of each study was extracted if accessible: response rate, clinical activity score, proptosis, diplopia, lid aperture/width, visual acuity, and side effects. And the characteristics or other important information was also recorded if possible: the title, authors, study design, publication year, location, inclusion and exclusion criteria, measurement point, measure methods of the recorded outcomes, and the definition of response rate mentioned in the paper. In addition, interventions, patient age, and sex as well as the number of patients lost were also included in the customized form. We estimated the data from the graphs by the software Plot Digitizer (version 2.6.8) if exact data were not accessible in the article.

### 2.5. Qualitative Assessment

The quality of included studies was appraised and described by two reviewers via a table that contained the influence factors of the bias. The qualitative assessment system was as follows: (1) allocation generation; (2) allocation concealment; (3) blinding of participants, investigators, and examiners; (4) the number of the patients lost to follow-up; (5) intension-to-treat (ITT) analysis; (6) selective reporting as described by the Cochrane Handbook; (7) other factors which would impact the bias of studies such as the equality of baseline of groups in the studies.

### 2.6. Statistical Analysis

We used the Review Manager software (RevMan, version 5.3) to conduct the statistical analysis. Risk ratio (RR) was calculated for the dichotomous variables (i.e., response rate) and mean difference (MD) for the continuous variables (i.e., proptosis) and standardized mean difference (SMD) for CAS because different clinical activity score systems were used in different trials, with 95% confidence intervals (CI). The mixture of the change-from-baseline and final value scores was included for proptosis because when using the (unstandardized) mean difference method in RevMan, it would not cause statistical problems. If any of the final value scores of CAS in the trials was unavailable in the same subgroup, the change-from-baseline value would be adopted to compare in this subgroup. When the baseline of outcome was unequal, the change-from-baseline score was also used to correct the bias. For each contrast, we estimated the heterogeneity by *χ*^2^ test and I^2^ metrics, and P < 0.1 or I^2^> 50% indicated the significant heterogeneity, in which case we would search for the reasons for obvious heterogeneity and chose a random effects model to analyze the combined results; otherwise we chose the fixed effects model. We estimated the mean and standard deviation (SD) through the data of median and range if necessary using the method reported by StelaPudarHozo, etc [6]. We included the data of the worse one if both sides of eyes were measured separately in the study.

## 3. Results

Twenty-nine trials were included in our meta-analysis. The selection process was shown in the flow diagram (S2 Diagram). And the characteristics of RCTs are summarized in Table 1. Patients of included studies had active GO in twenty-three trials, moderate to severe GO in twenty trials, and severe GO in one trial. The quality assessment of included studies is presented in Table 2. It should be noticed that patients in study Kahaly1986 were assigned on the basis of the year of birth. The adverse events and additional treatment during follow-up period are summarized in S3 Side-effects. The results would be presented by different interventions as follows.

### 3.1. Corticosteroids vs. Placebo

Two studies [7, 8] compared corticosteroids with placebo or control. Treatment with corticosteroids showed better curative effects in response rate; the pooled RR is 1.72 (95%CI: 1.28~2.31, P=0.0003), with heterogeneity (I^2^=63%). Methylprednisolone was administered intravenously to active moderately severe GO patients in the study van Geest2008 [8], which also proved marginal effects on reduction of CAS (95% CI: −2.27~-0.00), but no obvious effects on proptosis. Subconjunctival triamcinolone injections were administrated to inactive GO patients in study Lee2012 [7]. There were no major events during corticosteroid treatment (S3 Side-effects). 19 and 7 additional treatments were needed in placebo and corticosteroids group, respectively, during follow-up period.

### 3.2. Corticosteroids vs. Other Nonsurgical Therapy

Eight studies compared corticosteroids alone with other nonsurgical therapy including radiotherapy [9], rituximab [10, 11], cyclosporine [12], colchicine [13], immunoglobulin [14], mycophenolate mofetil (MMF) [15], and somatostatin [16]. And except for the rituximab, all of them reported the response rate.

**Figure 1:**
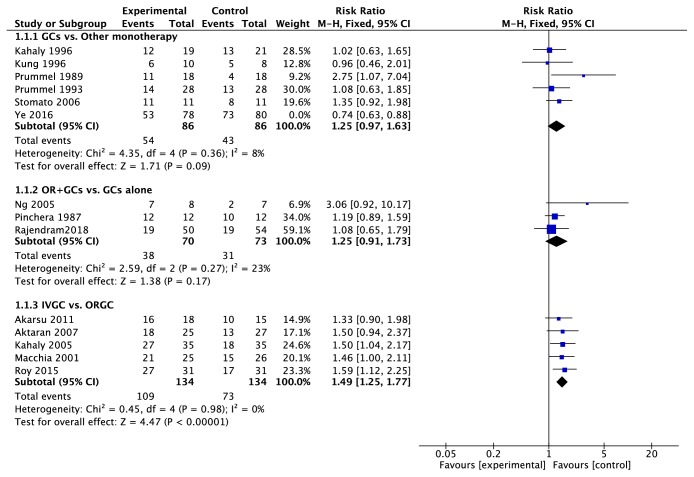
**Forest plot of the response rate. **The study Ye2016 was excluded when we combined the trails and its weight was 0% in the figure. SD: standard deviation. GCs: glucocorticoids. IVGC: intravenous injection of glucocorticoids. ORGC: oral glucocorticoids.

**Figure 2:**
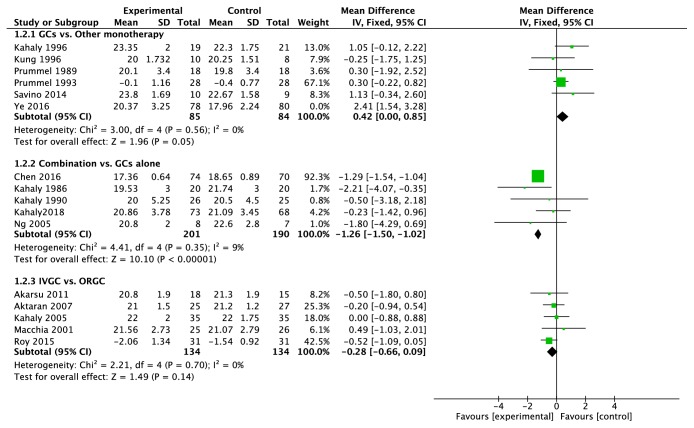
**Forest plot of proptosis. **The study Ye2016 was excluded when we combined the trails and its weight was 0% in the figure. SD: standard deviation. GCs: glucocorticoids. Combination: the combination of GCs treatment with another therapy. IVGC: intravenous injection of glucocorticoids. ORGC: oral glucocorticoids.

The sensitive analysis indicated that the study Ye2016, [15] which compared the methylprednisolone with MMF, increased the I^2^ value of heterogeneity from 8% to 69%. The MMF performed better in response rate (RR = 0.74, 95%CI: 0.63~0.88, P = 0.0005) (Figure 1) and reduction of CAS and proptosis (Figure 2) compared with GCs. On the contrary, the response rate of GCs was similar to immunoglobulin, colchicine, somatostatin, and radiotherapy and better than cyclosporine; in addition, GCs did not work better in proptosis reduction (MD = 0.42, 95%CI = 0.00~0.85, P = 0.05) (Figure 2).

Compared with systematic methylprednisolone treatment (total 4.5g), rituximab local injections did not work better in CAS or proptosis reduction in one study [11]. However, in another study, [10] systematic rituximab treatment was more effective in reduction of CAS than methylprednisolone treatment (total 7.5g) (SMD = 0.78, 95%CI: 0.05~1.52).

### 3.3. Combined Therapy vs. Monotherapy

Corticosteroid was combined with cyclosporine, [17] ciamexone, [18] technetium-99 methylene diphosphate (^99^Tc-MDP) [19], mycophenolate [20], or radiotherapy [21–23]. The result indicated that the combination of radiotherapy did not show extra effects compared with GCs alone (response rate, RR=1.25, 95%CI: 0.91~1.73, P=0.17) (Figure 1). On the contrary, combination of mycophenolate improved the response rate (RR=1.47, 95%CI: 1.09~2.00, P=0.01) and ^99^Tc-MDP or cyclosporine improved the proptosis (P<0.00001 and P=0.02).

### 3.4. Ideal Regimen of Corticosteroids Therapy

#### 3.4.1. Intravenous Corticosteroids vs. Oral Corticosteroid

**Figure 3:**
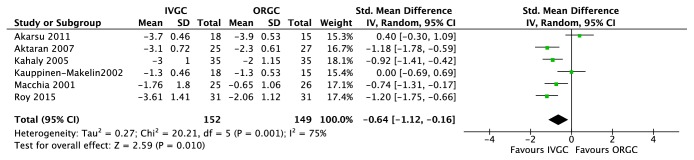
**Forest plot of clinical activity score. **SD: standard deviation. GCs: glucocorticoids. IVGC: intravenous injection of glucocorticoids. ORGC: oral glucocorticoids.

Six trials [24–29] compared intravenous glucocorticoids (IVGC) with oral glucocorticoids (ORGC) alone. IVGC were significantly better than ORGC in improvement of response rate (RR=1.49, 95%CI: 1.25~1.77, P<0.00001) (Figure 1) and CAS (SMD=-0.64, 95%CI: −1.12~-0.16, P=0.010) (Figure 3). There were six major adverse events recorded in oral group and none in the IVGC group (S3 Side-effects). And for the proptosis, there were no significant differences between two groups (MD = -0.28, 95% CI: −0.66~0.09, P = 0.14) (Figure 2).

#### 3.4.2. Different Doses and Protocols

Three regimens were mentioned in two trials [30, 31]: (1) monthly: 0.5g daily for 3 consecutive days in weeks 1, 5, 9, and 13 for a total dose of 6.0g over 3 months; (2) weekly: 0.5g weekly for 6 weeks, followed by 0.25g weekly for 6 weeks for a total dose of 4.5g over 12 weeks; (3) daily: 0.5g daily for 3 consecutive days per week for 2 weeks, followed by 0.25g daily for 3 consecutive days per week for another 2 weeks and by tapering oral prednisone. Weekly protocol was more effective than daily protocol and showed less adverse events than the other two protocols. Another trial [32] compared three different cumulative dosages of GCs and higher cumulative dosage (7.47g) provided a transient advantage. But considering the greater toxicity of higher dose, the intermediate-dose regimen (4.98g) was recommended.

#### 3.4.3. Others

Of the remaining three RCTs, one of them [33] compared the IVGC plus radiotherapy with ORGC plus radiotherapy, of which the result affirmed the advantage of IVGC against ORGC. Another trial [34] compared ORGC with peribulbar triamcinolone acetonide injection, which showed comparable effects. The other [35] proved the efficacy of dexamethasone instead of methylprednisolone.

## 4. Discussion

Immunosuppressant drugs are often used to treat GO, with glucocorticoids being the most common choice in the past decades depending on its anti-inflammatory function. However, it is still a challenge for us to manage GO with GCs for the following reasons. First, if it will be beneficial to receive another immunomodulatory drug instead of GCs to manage GO which is not clear. Second, the regimen of GCs, ranging from the administration route and drug dosage to the drug administration time, varied from study to study. In the present study, we performed a meta-analysis entirely around the usage of GCs in GO including twenty-nine RCTs, to help in confronting the challenge mentioned above.

The RCTs confirmed the effect of GCs whether given systemically or by local route. Compared with the placebo or observation group, GCs group had better response rate. GCs decreased the activity of GO in active GO patients and improved the eyelid swelling and retraction in recent-onset inactive ones. And most importantly, GCs treatment reduced the need for additional treatment such as ophthalmologic surgery.

Various nonsurgical therapies such as radiotherapy, colchicine, immunoglobulin, somatostatin rituximab, MMF, and cyclosporine were compared with GCs treatment; however, most of them have similar or inferior effects except MMF. However, the obvious advantage of MMF over GCs was only proved by one study and needs more RCT to confirm it. Therefore, it is still reasonable to regard GCs as first-line treatment for active moderate to severe GO. Considering its side effects, the usage of GCs depends on the health condition of patients and should be monitored to avoid serious side effects especially on liver function, glycaemia, and mood disorder.

A part of GO patients was not responsive to GCs treatment or relapsed after the withdrawal of GCs. Thus, combined therapy was taken into consideration. The combination of radiotherapy and GCs was not superior to GCs alone according to the results. As mentioned above, the effect of GCs treatment in proptosis improvement is not obvious. Firstly, GCs did not alleviate the proptosis more obviously compared with the placebos or other nonsurgical therapies. Secondly, although the IVGC performed better than ORGC, there still was not significant difference between these two groups in the reduction of proptosis. Thus, if the proptosis is the main symptom of patient, the combination of ^99^Tc-MDP or cyclosporine may be taken into consideration with cautious control of side effects.

Corticosteroids can be administered orally, intravenously, or locally, but locally administered corticosteroids, like subconjunctival or retrobulbar injections, may result in injuries, need more operative skills, and were not proved to be more effective, so they are not recommended first. Intravenous injection of GCs worked better than oral GC in response rate and CAS improvement, in accordance with the result reported by previous meta-analysis [36–38], which may be ascribed to rapidly increased and higher concentration of GCs in blood. Furthermore, intravenous injection of GCs also keeps patients healthy for a longer time and results in less advanced events. The result did not change when combined with radiotherapy. Therefore, the treatment of intravenous GCs should be recommended for active moderate to severe GO patients as suggested by the consensus made by EUGOGO. A few trials explored the optimal regimen of GCs, and the intermediate-dose (cumulative doses of 4.98g) and weekly protocol were recommended which however need more evidence.

In addition, it will be valuable to carry out more trails to confirm the advantage of MMF against GCs because it was proved obviously superior to GCs no matter as monotherapy or combination with GCs.

Our meta-analysis was also limited by some factors especially the small number of included studies. We used response rate, CAS, and proptosis as outcome measures. However, some studies only reported part of the outcomes. The characteristics of included studies are shown in Table 1. Most subjects recruited in the trials were active moderate to severe GO patients; therefore, the result of our analysis should be more suitable for this kind of patients. The quality of trails is summarized in Table 2. All of the trials were randomized controlled trails, but study Kahaly1986 was assigned on the basis of the year of birth, which would contribute to the inadequate allocation and the bias of study. What is more, the different dosage of GCs between studies comparing the GCs with other monotherapies can also bring the bias. Last, the number of RCTs was too small to support another drug as substitution for GCs or to guide the ideal regimen of GCs.

## 5. Conclusion

Our meta-analysis confirmed the effects of GCs in the management of GO and intravenous GCs is proved to be better than oral GCs as ever reported. Combination of radiotherapy and GCs did not show extra effects compared with GCs alone. However, if proptosis is the main issue, combination of ^99^Tc-MDP or cyclosporine with GCs may be taken into consideration. Recently, there have not been any suitable drugs for substitution of GCs; however, the reported advantage of mycophenolate mofetil over GCs is noteworthy and needs more RCTs to confirm.

## Figures and Tables

**Table 1:** The characteristics of including studies.

**Study**	**Location**	**Length**	**Age**	**Sex ** **(M/F)**	**Stage**	**Prior treatment (n)**	**Treatment group**		**Control group**	
							**Intervention**	**N**	**Intervention**	**N**
van Geest2008	Utrecht	12	47.3	3/12	Active, moderately severe	Untreated	GCs	6	Placebo	9
Lee2012	Korea	6	43.5	12/83	Inactive	No GCs or OR	GCs	75	Observation	59
Salvi2015	Italy	6	51.1	5/26	Active, moderate to severe	No GCs or cell depleting therapy	GCs	16	Rituximab	15
Savino2014	Italy	5	56.8	6/13	Active, moderately severe	No GCs	GCs	10	Rituximab	9
Prummel1993	Netherlands	6	46.8	9/47	Moderately severe	Untreated	GCs	28	OR	28
Prummel1989	Netherlands	3	50.5	10/26	Severe	Untreated	GCs	18	Cyclosporine	18
Stamato2006	Brazil	3	42.4	8/14	Active, moderate to severe	Untreated	GCs	11	Colchicine	11
Kahaly1996	Germany	5	47.5	9/31	Active	GCs/irradiation: 20	GCs	19	Immunoglobulin	21
Ye2016	Nanjing	6	41.1	52/106	Active, moderate to severe	Immunosuppressive or radiotherapy: 0	GCs	78	Mycophenolate mofetil	80
Kung1996	Hong Kong	3	42.1	9/9	Moderately severe	NA	GCs	10	Octreotide	8
Kahaly1986	Germany	12	46.8	5/35	NOSPECS III-V	18 GCs, 3 irradiation, 1 cyclophosphamide	GCs+ cyclosporine	20	GCs	20
Kahaly2018	Germany and Italy	9	51.4	39/125	Active, moderate to severe	Immunosuppressive treatment: 0	GCs+ mycophenolate	73	GCs	68
Chen2016	China	3	33.6	26/70	Active	NA	GCs+^99^Tc-MDP	74	GCs	70
Kahaly1990	Germany	6	51.0	9/42	Active, NOSPECS II-VI	Steroids/radiation: 40	GCs+ ciamexone	26	GCs	25
Ng2005	Hong Kong	13	56.2	10/6 (1 died)	Active, moderate to severe	Untreated	GCs + OR	8	GCs	7
Pinchera1987	Italy	26	44	11/13	Active	NA	GCs + OR	12	GCs	12
Rajendram2018	UK	12	49.3	33/93	Active moderate-to-severe	Immunosuppressive or radiotherapy: 0	GCs + OR	50	GCs	54
Roy 2015	India	12	37.3	24/38	Active, moderate to severe	Untreated	iv GCs	31	oral GCs	31
Macchia2001	Italy	Treat after	43.6	11/40	NA	Untreated	iv GCs	25	oral GCs	26
Akarsu2011	Turkey	6	28.9	12/21	Active, moderately severe	NA	iv GCs	18	oral GCs	15
Aktaran2007	Turkey	3	42.7	24/28	Active, moderately severe	Untreated	iv GCs	25	oral GCs	27
Kahaly2005	Germany	3	50	21/49	Active, moderately severe	Untreated	iv GCs	35	oral GCs	35
Kauppinen-Makelin2002	Finland	3	46.3	2/31	Active, or proptosis or diplopia	NA	iv GCs + oral GCs	18	oral GCs	15
Marcocci2001	Italy	12	49	14/68	Active, severity: defined by ocular manifestations	GCs/ciclosporin/octreotide / surgery: 12	iv GCs + OR	41	oral GCs + OR	41
Alkawas2010	Egypt	6	NA	8/16	Active, proptosis	Untreated	oral GCs	12	GCs injection	12
Bartalena2012	8 EUGOGO centers	3	52.9	33/73	Active, moderate to severe	Untreated	IVMP (total 7.47g)	52	IVMP (total 4.98g)	54
He2016	China	3-3.25	41.8	14/26	CAS≥3/7 or prolonged T2RTs; Moderate to severe	Untreated	IVMP monthly (total 6.0g)	17	IVMP weekly (total 4.5g)	15
Zhu2014	China	3	46.8	34/46	Active, moderate to severe	Immunosuppressive or radiotherapy: 0	IVMP weekly	39	IVMP daily	39
Philip2013	India	3	37.5	5/16	Active, moderate to severe	NA	Dexa	11	IVMP	10

Length: the time when final values included in our analysis were measured and represented as the number of months. GCs: glucocorticoids; OR: orbital radiotherapy; IVMP: intravenous methylprednisolone; iv: intravenous; N: numbers. Dexa: dexamethasone.

**Table 2:** Methodological quality of randomized clinical trials included in the meta-analysis.

**Study**	**Allocation generation; ** **concealment**	**Binding**			**Follow-up lost (n)**	**ITT**	**Selective Reporting**	**Unequal baseline or other remarks**
		**Participants**	**Investigators**	**Examiners**				
van Geest2008	A, A	Y	N	Y	1 at week 0	Y	NA	
Lee2012	A, B	N	N	Y	10	N	N	Swelling grade
Salvi2015	A, B	Y	N	Y	1	N	Y	Protocol amendment
Savino2014	B, B	N	N	N	1	N	NA	
Prummel1993	A, B	Y	N	Y	3	N	NA	
Prummel1989	A, B	N	N	Y	0	Y	NA	
Stamato2006	A, B	Y	N	Y	3	N	NA	
Kahaly1996	A, B	N	N	Y	0	Y	NA	Visual acuity
Ye2016	A, B	N	N	Y	16	N	NA	
Kung1996	A, B	N	N	Y	0	Y	NA	CAS
Kahaly1986	C, C	N	N	Y	0	Y	NA	
Kahaly2018	A, A	N	Y	Y	23	N	N	
Chen 2016	A, B	N	N	N	0	Y	NA	
Kahaly1990	B, B	Y	N	Y	0	Y	NA	
Ng2005	B, B	N	N	Y	1	N	NA	Age
Pinchera1987	A, B	N	N	N	0	Y	NA	
Rajendram2018	A, B	Y	Y	Y	69	Y	N	Ethnicity
Roy2015	A, B	N	N	N	3	N	N	Diplopia, TSH
Macchia2001	B, B	N	N	N	0	Y	NA	CAS, OI
Akarsu2011	B, B	N	N	N	0	Y	NA	
Aktaran2007	A, A	N	N	Y	0	Y	NA	Lid width
Kahaly2005	A, B	N	N	Y	0	Y	NA	Lid width
Kauppinen-Makelin2002	A, A	N	N	N	0	Y	NA	TSab titers, visual acuity
Marcocci2001	A, B	N	N	Y	0	Y	NA	
Alkawas2010	B, B	N	N	N	5	N	NA	
Bartalena2012	A, A	Y	N	Y	6	Y	NA	Age, gender
He2016	A, B	N	N	Y	8	N	NA	
Zhu2014	A, A	N	N	N	2	Y	NA	Duration of eye symptoms; TRAb
Philip 2013	B, B	N	N	N	0	Y	NA	

ITT: intention to treat analysis; A: adequate; B: unknown; C: inadequate; N: no; Y: yes; NA: unable to assess; CAS: clinical activity score; TSH: thyroid stimulating hormone; OI: ophthalmopathy index score; TSab: thyroid stimulating antibodies; TRAb: TSH receptor antibody.

## Data Availability

The data used to support the findings of this study are included within the article.

## Supplementary Materials

Supplementary Materials
** S1 Strategy**: the search strategy.** S2 Diagram**: the selection process of eligible randomized controlled trails.** S3 Side-effects**: the adverse events and additional treatments during follow-up period.
